# Circadian Clock Protein Content and Daily Rhythm of Locomotor Activity Are Altered after Chronic Exposure to Lead in Rat

**DOI:** 10.3389/fnbeh.2017.00178

**Published:** 2017-09-22

**Authors:** Mariam Sabbar, Ouria Dkhissi-Benyahya, Abdelhamid Benazzouz, Nouria Lakhdar-Ghazal

**Affiliations:** ^1^Équipe de Recherche sur les Rythmes Biologiques, Neurosciences et Environnement, Faculté des Sciences, Université Mohammed V Rabat, Morocco; ^2^INSERM, Stem Cell and Brain Research Institute U1208, University of Lyon, Université Claude Bernard Lyon 1 Lyon, France; ^3^Institut des Maladies Neurodégénératives, Univ. de Bordeaux, UMR5293 Bordeaux, France; ^4^Centre National de la Recherche Scientifique, Institut des Maladies Neurodégénératives, UMR5293 Bordeaux, France

**Keywords:** suprachiasmatic nucleus, lead, locomotor activity, clock proteins, rat, Parkinsonism

## Abstract

Lead exposure has been reported to produce many clinical features, including parkinsonism. However, its consequences on the circadian rhythms are still unknown. Here we aimed to examine the circadian rhythms of locomotor activity following lead intoxication and investigate the mechanisms by which lead may induce alterations of circadian rhythms in rats. Male *Wistar* rats were injected with lead or sodium acetate (10 mg/kg/day, i.p.) during 4 weeks. Both groups were tested in the “open field” to quantify the exploratory activity and in the rotarod to evaluate motor coordination. Then, animals were submitted to continuous 24 h recordings of locomotor activity under 14/10 Light/dark (14/10 LD) cycle and in complete darkness (DD). At the end of experiments, the clock proteins BMAL1, PER1-2, and CRY1-2 were assayed in the suprachiasmatic nucleus (SCN) using immunohistochemistry. We showed that lead significantly reduced the number of crossing in the open field, impaired motor coordination and altered the daily locomotor activity rhythm. When the LD cycle was advanced by 6 h, both groups adjusted their daily locomotor activity to the new LD cycle with high onset variability in lead-intoxicated rats compared to controls. Lead also led to a decrease in the number of immunoreactive cells (ir-) of BMAL1, PER1, and PER2 without affecting the number of ir-CRY1 and ir-CRY2 cells in the SCN. Our data provide strong evidence that lead intoxication disturbs the rhythm of locomotor activity and alters clock proteins expression in the SCN. They contribute to the understanding of the mechanism by which lead induce circadian rhythms disturbances.

## Introduction

Lead poisoning has been reported to induce Parkinsonism and epidemiological studies suggested that lead plays a synergistic role with other heavy metals in the incidence of Parkinson's disease (PD) (Gorell et al., 1997, 1999a,b). In fact, the role that lead may play to generate Parkinsonism has been strongly supported by many studies demonstrating that this heavy metal is affecting the dopaminergic (DA) system as reported in PD (Ehringer and Hornykiewicz, 1960). Jason and Kellogg (1981) reported that neonatal exposure to lead in rats induced an irreversible degeneration of the nigro-striatal dopaminergic system in parallel with behavioral and neurochemical abnormalities (Jason and Kellogg, 1981). Other studies have shown that lead exposure in adolescent rat also decreased dopamine levels (Kala and Jadhav, 1995) and that this decrease may not be related to the loss of tyrosine hydroxylase immunoreactive neurons in the *pars compacta* of substantia nigra as reported by Tavakoli-Nezhad et al. (2001).

We have recently shown that sub-chronic exposure to lead acetate induced motor deficits in parallel with a decrease in the content of both DA in the striatum and noradrenaline (NA) in the cortex (Unpublished data).

Non-motor deficits, such as anxiety disorder have also been observed in the rat following lead exposure (Sabbar et al., 2012). Similar motor and non-motor disorders have been reported in PD patients (Shulman et al., 2001; Barone et al., 2009; Breen et al., 2014; Alzahrani and Venneri, 2015), in animal models of PD (Delaville et al., 2012; Faggiani et al., 2015) and in animals exposed to manganese (Bouabid et al., 2014). Interestingly, non-motor symptoms have been shown to precede the manifestation of motor disabilities in PD patients (Chaudhuri et al., 2006; Ishihara and Brayne, 2006). Furthermore, one of the common non-motor disorders reported in PD patients is sleep disorder, including insomnia, excessive daytime sleepiness and disturbed rapid eye movement (REM) sleep behavior (Gunn et al., 2010; Menza et al., 2010; Willison et al., 2013).

The sleep-wake cycle is one of the many daily rhythms regulated by the circadian timing system (Pace-Schott and Hobson, 2002). Circadian rhythms are controlled by the master clock localized in the suprachiasmatic nuclei (SCN) of the anterior hypothalamus in mammals (Stephan and Zucker, 1972). The circadian clock generates molecular circadian rhythms through cell-autonomous autoregulatory transcriptional/translational feedback loops consisting of the bHLH/PAS transcription factors BMAL1 and CLOCK, which heterodimerize and drive transcription of many genes, including their own negative feedback repressors, such as *Period* (*Per1, Per2, Per3*), *Cryptochrome* (*Cry1, Cry2*) and *Reverb* genes, which repress BMAL1/CLOCK-mediated transcription (Reppert and Weaver, 2001; Lowrey and Takahashi, 2004; Okamura, 2004).

The consequences of lead exposure on the circadian system have been investigated by few studies who showed that lead exposure affected the locomotor activity rhythm in rats (Collins et al., 1984). Using the rat model of lead-induced Parkinsonism, the present study aimed to investigate whether lead exposure affects both the circadian rhythms of locomotor activity and the molecular machinery of the SCN. The effects of a sub-chronic low-level lead treatment on motor behavior and coordination was first evaluated; then, we monitored the daily and circadian locomotor activity rhythm and quantified clock proteins expression; BMAL1, PER1, PER2, CRY1, and CRY2 in the SCN.

## Materials and methods

### Animal housing

Male *Wistar* rats (Central animal service, Mohammed V University, Faculty of Sciences, Rabat, Morocco) weighing 70–80 g were used for behavioral and immunohistochemical studies. Rats were kept individually in polycarbonate cages, in a thermostatically controlled room (temperature: 24°C, relative humidity: 45%) under a 14 h/10 h light/dark cycle (14/10 LD; lights on at 06:00 h) for 3 weeks, with access to food and water *ad libitum*. Body weights were monitored throughout the experiment. All experiments were performed in accordance with the European Communities Council Directive 2010/63/UE. Approval was granted by the Ethic Committee of Veterinarians of Hassan II Institute of Agronomy and Veterinary Medicine of Rabat, Morocco, and all efforts were made to minimize the number of animals used and their suffering.

### Lead administration

Animals were randomly divided into two groups: lead-intoxicated (*n* = 26) and control (*n* = 20) rats. In our experiments, lead-intoxicated rats received daily i.p. injections of lead acetate free-pyrogen solution (10 mg/kg; Sigma, France) at zeitgeber time 4 (ZT4, 4 h after light on), for 30 days. Control rats received sodium acetate (10 mg/kg; Sigma, France) solution in the same conditions.

### Behavioral assessments

#### Assessment of spontaneous locomotor activity

Exploratory behavior was evaluated in the open field test as previously described by Rodrigues et al. (1996) with modification. The apparatus is a wooden box (75 cm long, 45 cm wide and 35 cm high), the surface was divided into 15 similar squares of 15 cm each side. Prior to lead or sodium acetate administration, all rats were habituated to the open field for 3 days. At the first and the last day of the i.p. injections of lead or sodium acetate, rats were tested once in a quiet and dimly lit for 5 min session. Exploratory behavior was evaluated by counting the number of crossing (transition from one square to another) and rearing (animal stands on its two legs).

#### Assessment of motor coordination

Motor coordination was evaluated using the rotarod test. The apparatus is equipped with a rotating bar which rotates at different and adjustable speeds. Rats were placed on the rotating bar with a fixed speed of 20 rotations per minute (rpm). The performance on the rotarod was measured once a day at ZT4 (10:00 h) for 4 consecutive days, and the time was recorded until the rat failed to stay on the bar.

#### Assessment of locomotor activity rhythm

Locomotor activity was continuously monitored using infrared motion captors placed over the cages and a computerized data acquisition system CAMS (Circadian Activity Monitoring System, INSERM, France) as previously described (El Moussaouiti et al., 2010), and analyzed using Clocklab software (Actimetrics, Evanston, Illinois, USA). Different parameters were analyzed: the period and the amplitude using Chi-squared periodogram, daytime and nighttime activities, onset and offset variabilities, activity onset and offset which correspond to the average clock (or circadian) time of activity onset or activity offset respectively. The diurnality index [mean activity during the light phase/(mean activity during the light phase + mean activity during the dark phase)] as previously described (Refinetti, 2006). Animals with indices above 0.5 are more active during the day than during the night. Alpha (α) was also analyzed and is defined as the duration of the active period of the animal. To assess the entrainment of the rhythm of locomotor activity by light, animals were subjected to 6 h phase advance of the 14/10 LD cycle (lights on at 00:00 h; LD AT) for 3 weeks after the last injection of either lead or sodium acetate. The phase angle and the number of days necessary to entrain to the new 14/10 LD cycle were determined for each rat. The phase angle was defined as the difference between the onset activity and the time of lights-off. In our study, the rhythm of locomotor activity is considered entrained to the new LD cycle when the onset of activity presents a stable phase relationship relative to the time of light off (±0.3 h) for at least 10 days as previously described (Lahouaoui et al., 2014).

Recording of the circadian locomotor activity was pursued in constant darkness (DD) for 15 days, and the free-running periods (Tau, τ) were calculated using Clocklab (Actimetrics, Evanston, Illinois, USA). Then, rats were exposed to 14/10 LD cycle (lights on at 06:00 h) for 10 days.

#### Experimental procedures

**Figure 1:**
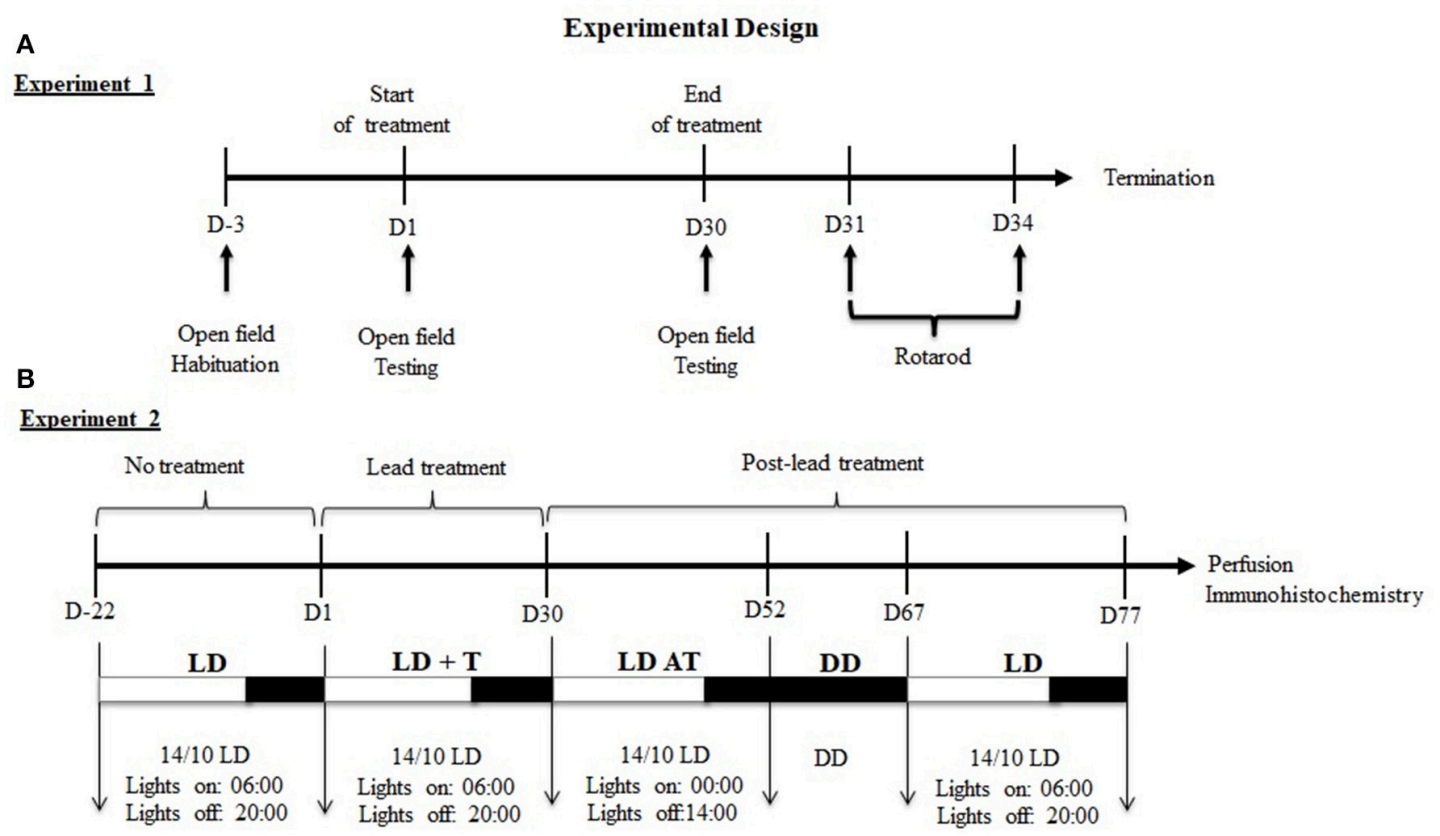
Experimental design of experiments 1 and 2. **(A)** Experiment1: Sequence of behavioral tests. **(B)** Experiment2: Sequence of lighting protocols and the duration of each cycle. Under LD cycle, locomotor activity was recording before (LD, 3 weeks), during (LD+T, 4 weeks) and after lead treatment (LDAT, 3 weeks). Locomotor activity was recorded in total darkness (DD) for 15 days.

***Experiment 1:*** Prior to lead or sodium acetate administration, all rats were maintained under 14/10 h LD cycle, with lights on at 06:00 h and were habituated to the open field for 3 days. On day1 (Figure 1A), rats were assigned randomly to receive an i.p. injections of one of the following substances: lead acetate (10 mg/kg; *n* = 6), and sodium acetate (10 mg/kg; *n* = 6) at ZT 4 (10:00 h) for 30 days. On day 30, after the last injection of lead or sodium acetate, a rat was placed into the open field box and allowed to explore the box for about 5 min. Exploratory behavior was evaluated by counting the number of crossing (transition from one square to another) and rearing (animal stands on its two legs).

Twenty-four hours after the open field test, the performance on the rotarod was evaluated in the same rats. Rats were placed on the rotating bar with a fixed speed of 20 rpm. The performance on the rotarod was measured once a day at ZT4 for 4 consecutive days, and the time was recorded until the rat failed to stay on the bar (Figure 1A).

***Experiment 2***: Rats were maintained under 14/10 h LD cycle, with lights on at 06:00 and daily locomotor activity rhythms of rats were recorded for 22 days, prior lead or sodium acetate treatment (LD; Figure 1B). On day 1, rats were assigned randomly to receive an i.p. injection of one of the following substances: lead acetate (10 mg/kg; *n* = 6), sodium acetate (10 mg/kg; *n* = 6) at ZT 4 (10:00 am) for 30 days and were maintained under 14/10 h LD cycle (LD+T; Figure 1B). On day 30, after the last injection of lead or sodium acetate, rats were subjected to 6 h phase advance of the 14/10 LD cycle (lights on at 00:00 h; LD AT) to examine the ability of animals to re-entrain to a new LD cycle for 22 days (Figure 1B). Rats underwent under total darkness (DD; Figure 1) to assess the circadian locomotor activity rhythm for 14 days followed by 10 days in 14/10 LD cycle (light on at 06:00 h).

### Immunohistochemistry

At the end of the locomotor activity recording period, rats were deeply anesthetized by an injection of sodium pentobarbital (100 mg/kg, i.p.) and perfused with 0.9% saline followed by 4% paraformaldehyde (PFA, 300 ml) in 0.1 M phosphate buffer (PB, pH 7.4). Brains were removed and post-fixed in 4% PFA at 4°C for an additional 24 h, rinsed in PBS, and cryo-protected in 30% sucrose in 0.1 M PBS (phosphate-buffered saline, pH 7.4) for additional 24 h at 4°C. Collected brains from control (*n* = 12) and lead-intoxicated (*n* = 12) rats were cut into 20 μm coronal sections and two series of three alternated sections were collected. We used one of the series for the primary antibodies; anti-PER1, anti-PER2 and anti-BMAL1, and the second serie for the primary antibodies; anti-CRY1 and anti-CRY2.

SCN sections were pre-incubated in 0.1 M PBS, 5% normal goat serum with 0.4% Triton X-100 and 1% bovine albumin serum for 1 h and then transferred to 2% normal goat serum containing primary antibody for 48 h at 4°C (1:5,000). After washes in 0.1 M PBS (3 × 10 min), sections were incubated during 2 h in 0.1 M PBS containing 2% normal goat serum and biotinylated rabbit anti-goat IgG (1:2,000, Vector laboratories) for 2 h at room temperature. Sections were then washed in PBS (3 × 10 min), and incubated with the avidin/biotin complex (1:1,000; ABC, Vectastain kit, Vector Laboratories) for 1 h at room temperature. After several washings (two in 0.1M PBS and one in 0.05 M Tris buffer (TB, pH 7.6), immunoreactivity was visualized with 0.025% DAB (Sigma, France), 0.5% ammonium nickel sulfate (Sigma, France), in 0.1 M TB containing 0.03% hydrogen peroxide (Sigma, France), for 6–10 min. Sections were finally washed three times in 0.1 M TB and once in 0.1 M PB. After processing, tissue sections were mounted onto gelatin-coated slides, dehydrated in graded ethanol, cleared in xylene and coverslipped with Eukitt (Sigma, France).

### Data analysis

All Statistical analyses (behavioral or immunohistochemical analyses) were done using GraphPad Prism program version 6.05 (California, USA). Data are shown as mean ± SEM and statistical significance was considered for *P* < 0.05.

Two-way ANOVA followed by *post-hoc* Bonferroni were used to compare body weight, number of crossing and rearing, response latency on the rotarod (failure time) between the two groups. Period, tau, activity onset and offset variabilities, α, phase angle, and diurnality index were compared using Mann-Whitney test between controls and lead-intoxicated rats. Mean activity counts during the light and the dark phases and the total activity were also analyzed using Mann-Whitney test.

Labeled cell nuclei by PER 1-2, CRY1-2, and BMAL1 were counted manually in 4–5 sections per animal for each primary antibody) of the SCN using a computerized image system (Image J software, imagej.nih.gov/ij/) attached to a light microscope (Leica Microsystems, Germany). The number of immunoreactive cells (ir-cells) in the SCN was counted in each section and averaged among these coronal sections. Statistical analysis was determined using the Mann-Whitney test.

## Results

### Lead intoxication decreased body weight gain

**Figure 2:**
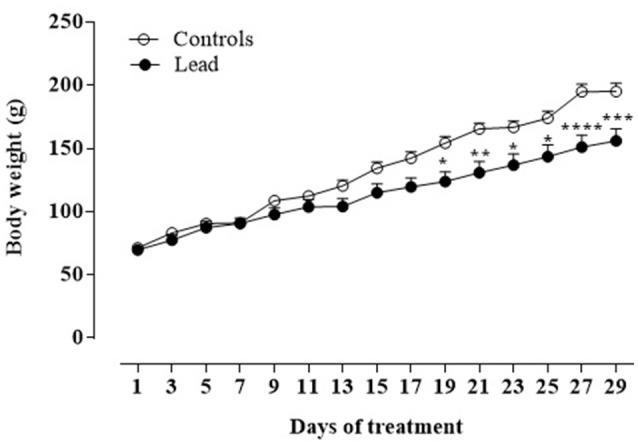
Effect of lead treatment on body weight gain. Change in body weight (mean ± SEM) of rats during lead treatment. Body weight was measured every 3 days. Data from controls (*n* = 6) and lead-intoxicated rats (*n* = 8) were compared using Two-way ANOVA followed by Bonferroni *post-hoc* test. ^*^P < 0.05, ^**^P < 0.001, ^***^P < 0.001, ^****^P < 0.0001 in comparison with controls.

Figure 2 shows the mean body weight (±SEM) of lead-intoxicated rats and their respective controls. Two-way repeated measures ANOVA showed significant effects of lead on time [*F*_(14, 168)_ = 294.6, *P* < 0.0001], treatment [*F*_(1, 12)_ = 5.672, *P* < 0.05] and interaction (treatment × time) [*F*_(14, 168)_ = 13.57, *P* < 0.0001]. At the beginning of the experiment, all animals had similar body weights (lead-intoxicated rats: 69.75 ± 3.22 vs. control rats: 71.33 ± 2.39). From day 19 to day 29 of injections, *post-hoc* test revealed that body weight of lead-intoxicated rats became significantly reduced compared to controls and resulted in 23% loss of body weight gain on day 29 (Bonferroni *post-hoc* test, *P* < 0.001; Figure 2).

### Lead intoxication induced hypoactivity measured in the open field test

In the open field test, we expected that exploratory activity will be higher independently to the treatment in day 30, because the age of the rat influences the exploration rate; infant or adolescent rats had less exploratory activity than adults (Smith and Morrell, 2007). We focused, then in our data analysis in the effect of lead intoxication on the exploratory activity on day 30 compared to their controls on the same test day.

**Figure 3:**
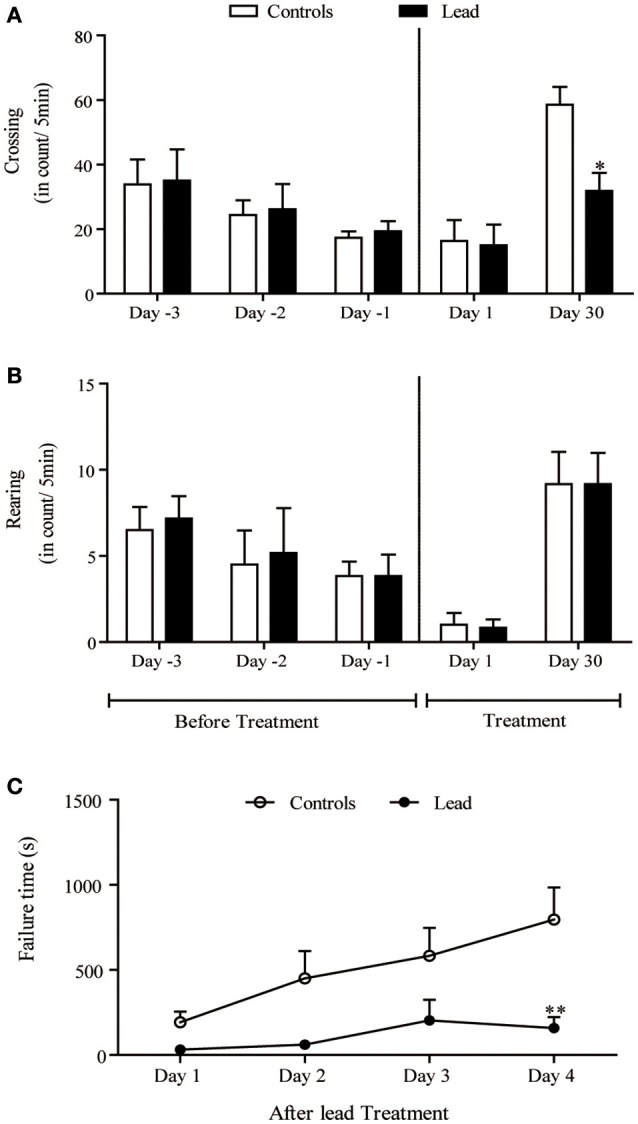
Lead treatment reduced locomotor activity and disturbed motor coordination Histograms of exploratory activity represent the number of crossing **(A)** and rearing **(B)** measured in the open field test during 5 min sessions on the first day and the last day of lead acetate treatment. Motor coordination performance histogram represent the time that rat stayed on the rotating bar using the rotarod test at the end of lead treatment **(C)**. Values are the mean ± SEM. Data from controls (*n* = 6) and lead-intoxicated rats (*n* = 6) were compared using two-way ANOVA followed by a *post-hoc* Bonferroni. ^*^P < 0.05, ^**^P < 0.01 in comparison with controls.

Figure 3 shows the spontaneous locomotor activity (crossing; Figures 3A,B) as measured in the open field test. There were no statistically significant differences among the groups in the number of crossing and rearing movements (Figures 3A,B) on the habituation period before injections, as well as on the first day of injections.

Upon a re-introduction into the open field on day 30, the pattern of the exploratory activity in rats was notably different; the number of crossings significantly decreased in lead-intoxicated rats compared to controls [−45.59%, *F*_(4, 40)_ = 3.20, *P* < 0.05, two-way ANOVA followed by Bonferroni *post-hoc* test; Figure 3A]. However, the number of rearings did not significantly change [*F*_(4, 40)_ = 0.040, *P* > 0.05, two-way ANOVA, Figure 3B].

### Lead intoxication impaired motor coordination evaluated in the rotarod test

The motor coordination was assessed using the rotarod test. Latency time in seconds was recorded. Two-way repeated measures ANOVA of motor coordination after lead acetate or sodium acetate injections showed a significant effects on time [*F*_(3, 30)_ = 9.400, *P* = 0.0002], and treatment [*F*_(1, 10)_ = 6.932, *P* = 0.0250] but not on treatment × time interaction [*F*_(3, 30)_ = 2.802, *P* = 0.0568]. Lead-intoxicated rats did not significantly increase their latency time on the rotating bar, in contrast to controls, in which the latency time on the rotating bar increased significantly (Bonferroni *post-hoc* test, *P* < 0.01, Figure 3C).

### Lead intoxication disturbed the daily and the circadian locomotor activity rhythm

**Figure 4:**
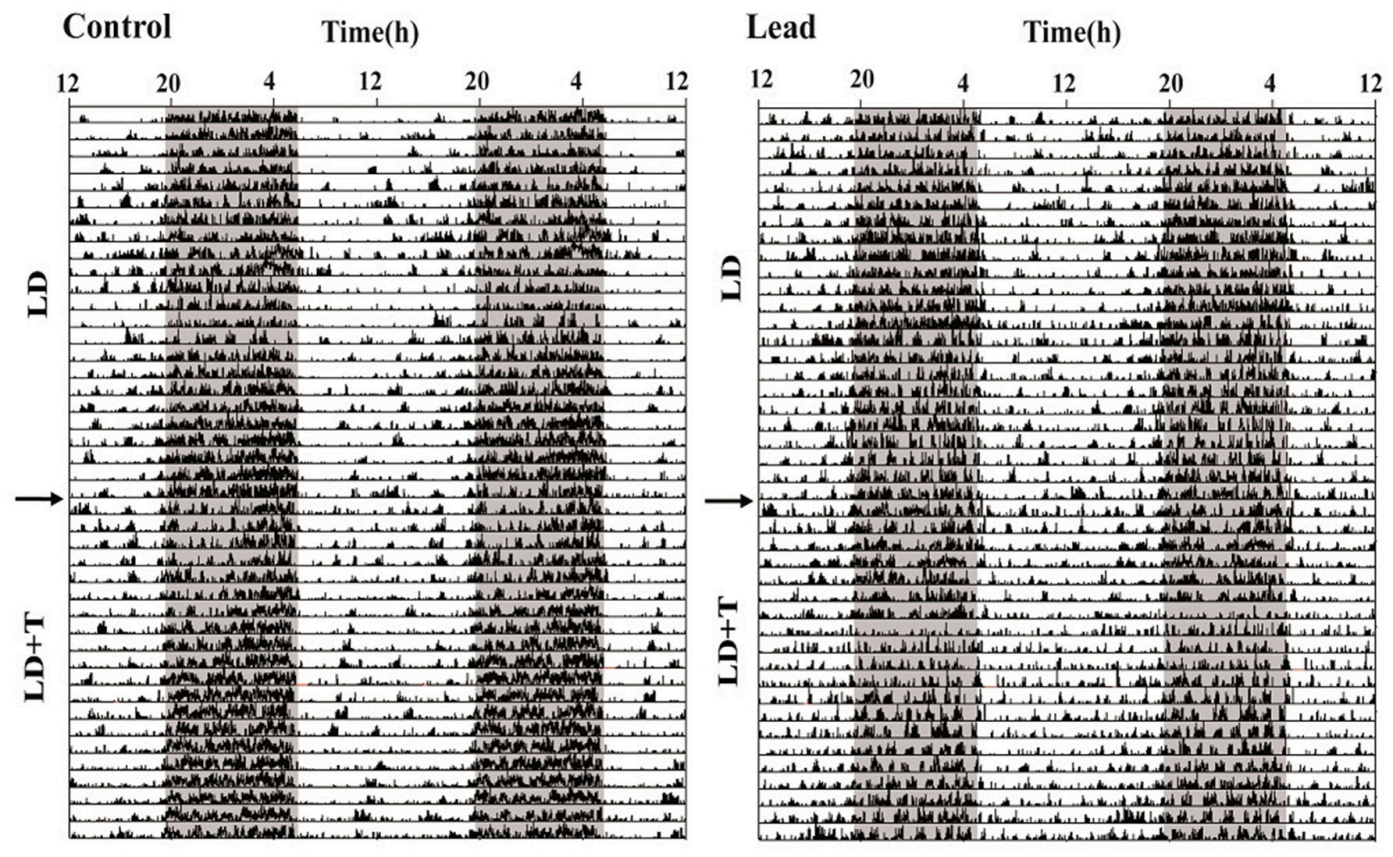
Representative double-plotted actograms illustrating locomotor activity rhythm of a control rat and lead-intoxicated rat. The horizontal axis represents clock time (h), with 48 h of data shown on each row, while the vertical axis represents study days. The gray bar represents the dark phase and white bar represent the light phase. The arrow indicates the beginning of lead or sodium acetate treatment.

As mentioned earlier (see section Material and Methods), all rats were randomly divided into two groups; controls and lead-intoxicated rats. The daily locomotor activity rhythm of all rats was monitored when animals were exposed to 14/10 LD cycle prior to any treatment. Each animal served as its own control. Representative actograms before treatment (LD), and during lead or sodium acetate injections (LD+T) are shown in Figure 4.

In LD, during the pre-treatment, all rats showed a strong daily profile of locomotor activity with the main activity was recorded during the dark phase. The average daytime activity counts was 447.1 ± 29.34 and the average nighttime activity counts was 1,390 ± 94.28. After this pre-treatment rats, we divided randomly all rats to two groups lead-intoxicated and control group and the data were re-analyzed and no significant differences were observed in the mean activity.

**Figure 5:**
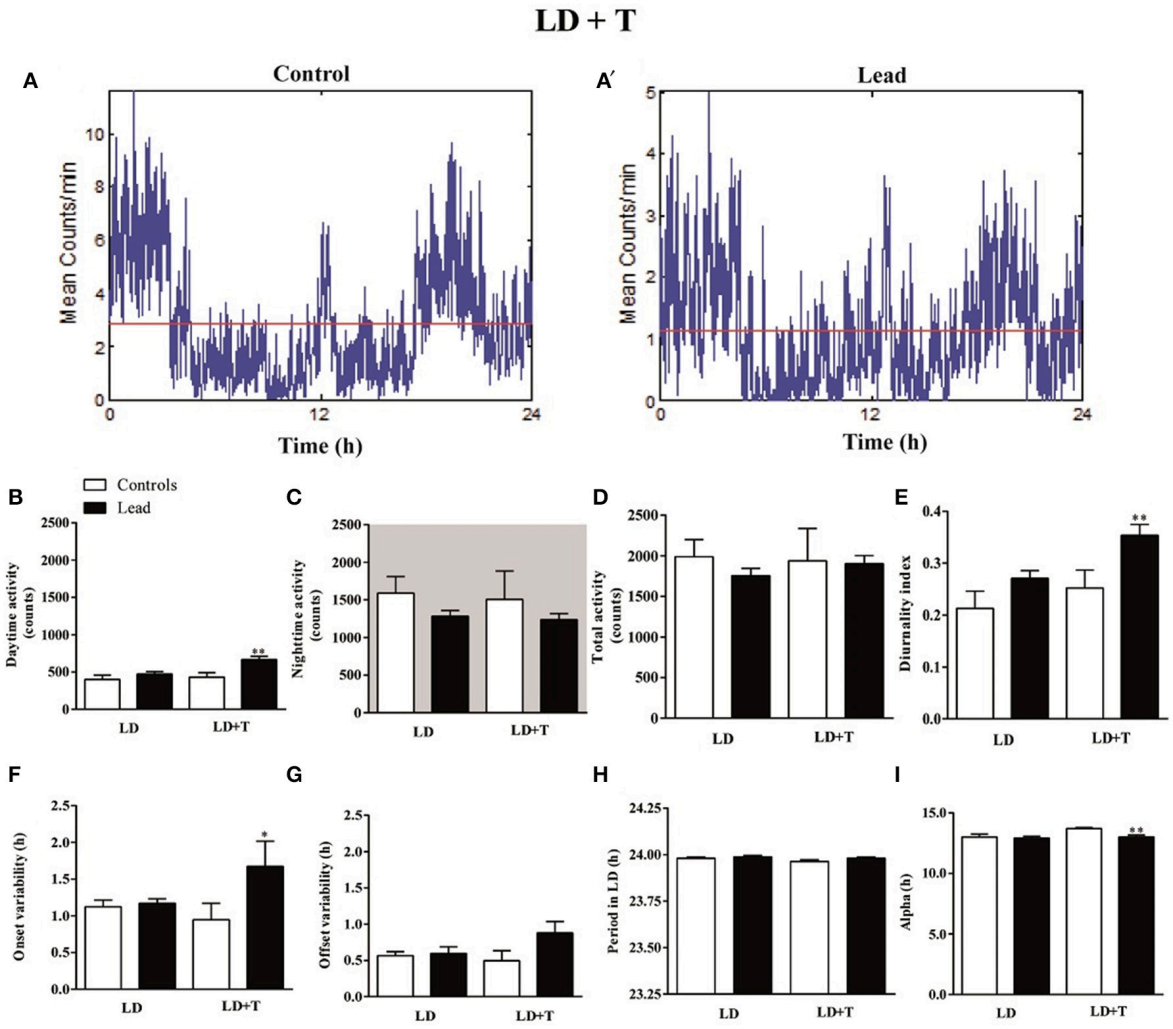
Circadian parameters for controls and lead-intoxicated rats in LD before (LD) and during lead or sodium acetate treatment (LD+T). **(A)** Activity profile illustrates the 24 h activity in LD cycle in lead-intoxicated rat compared to control rat. **(B)** Daytime activity counts, **(C)** Nighttime activity counts, **(D)** Total activity counts, **(E)** Diurnality index, **(F)** Period **(G)** Onset variability, **(H)** Offset variability, and **(I)** Alpha. Data from controls (*n* = 7) and lead-intoxicated rats (*n* = 13) were compared using Mann-Whitney test. ^*^P < 0.05, ^**^P < 0.01 in comparison with controls.

The activity profile for representative control and lead-intoxicated rats are shown in Figure 5A. During lead intoxication (LD+T), daytime activity counts were significantly increased in lead-intoxicated rats when compared to their controls (666.8 ± 43.14 vs. 430.6 ± 61.68 in controls *P* < 0.01, Mann Whitney test, Figure 5B). However, no significant differences were found between lead-intoxicated rats and controls in the nighttime activity counts (1,236 ± 83.03 vs. 1,506 ± 376.5 in controls, Figure 5C) or in the total activity counts (1,903 ± 100.5 vs. 1,937 ± 399.2 in control rats, Figure 5D). The diurnality index was significantly higher in lead-intoxicated rats (0.35 ± 0.02) compared to controls (0.25 ± 0.03) (*P* < 0.01, Mann Whitney test; Figure 5E).

Periodogram analysis showed that both groups remained entrained to the 14/10 LD cycle during lead intoxication (LD+T), with similar period (23.96 ± 0.009 h in controls vs. 23.98 ± 0.006 h in lead-intoxicated rats; Figure 5F) but with a delay in their activity onset. The nighttime activity began in lead-intoxicated rats before light OFF (onset variability was 0.94 ± 0.22 h in controls vs. 1.67 ± 0.34 h in lead-intoxicated rats; *P* < 0.05, Mann Whitney test; Figure 5G). No difference in the offset of activities was observed (offset variability was 0.49 ± 0.13 h in controls vs. 0.88 ± 0.16 h in lead-intoxicated rats; *P* = 0.054, Mann Whitney test, Figure 5H). α significantly decreased in lead-intoxicated rats with a mean value of 13.01 ± 0.16 h by comparison to controls (13.71 ± 0.08 h; *P* = 0.0034, Mann Whitney test, Figure 5I).

One day after the last injection of lead or sodium acetate, rats have been exposed to a 6 h phase advance of the 14/10 LD cycle to examine their ability to entrain to a new LD cycle.

**Figure 6:**
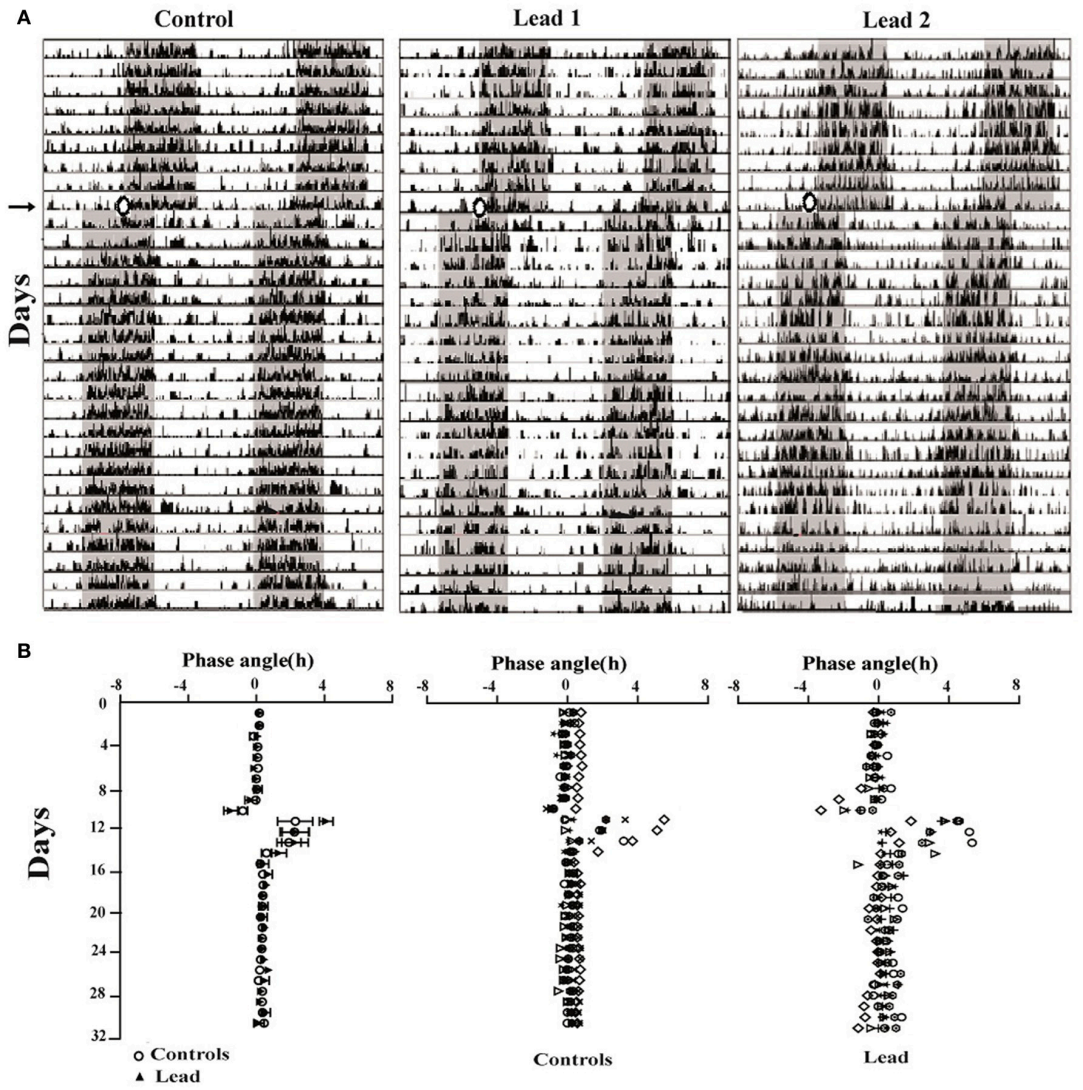
Lead treatment did not affect photic entrainment. **(A)** Three double-plotted actograms of locomotor activity for control and two lead-intoxicated rats under LD cycle (LD + T. → LD AT). The horizontal axis represents time (h), with 48 h of data shown on each row, while the vertical axis represents study day. The gray bar represents the dark phase and white bars represent the light phase. **(B)** Phase angles of activity onsets showing the low onset precision after the entrainment to the new LD (**LDAT**) cycle for all rats (Mean ± SEM) and individual data from controls and lead-intoxicated rats.

Representative actograms from control and lead-intoxicated rats are shown in Figure 6A. Control and lead-intoxicated rats were able to synchronize their locomotor activity rhythm to the new LD cycle. In controls, entrainment was achieved after 8.67 ± 1.17 days, whereas in lead-intoxicated rats, the entrainment was attained after 8.85 ± 1.81 days. No significant difference between the two groups was observed (Mann Whitney test, *P* = 0.80, Figure 6B).

**Figure 7:**
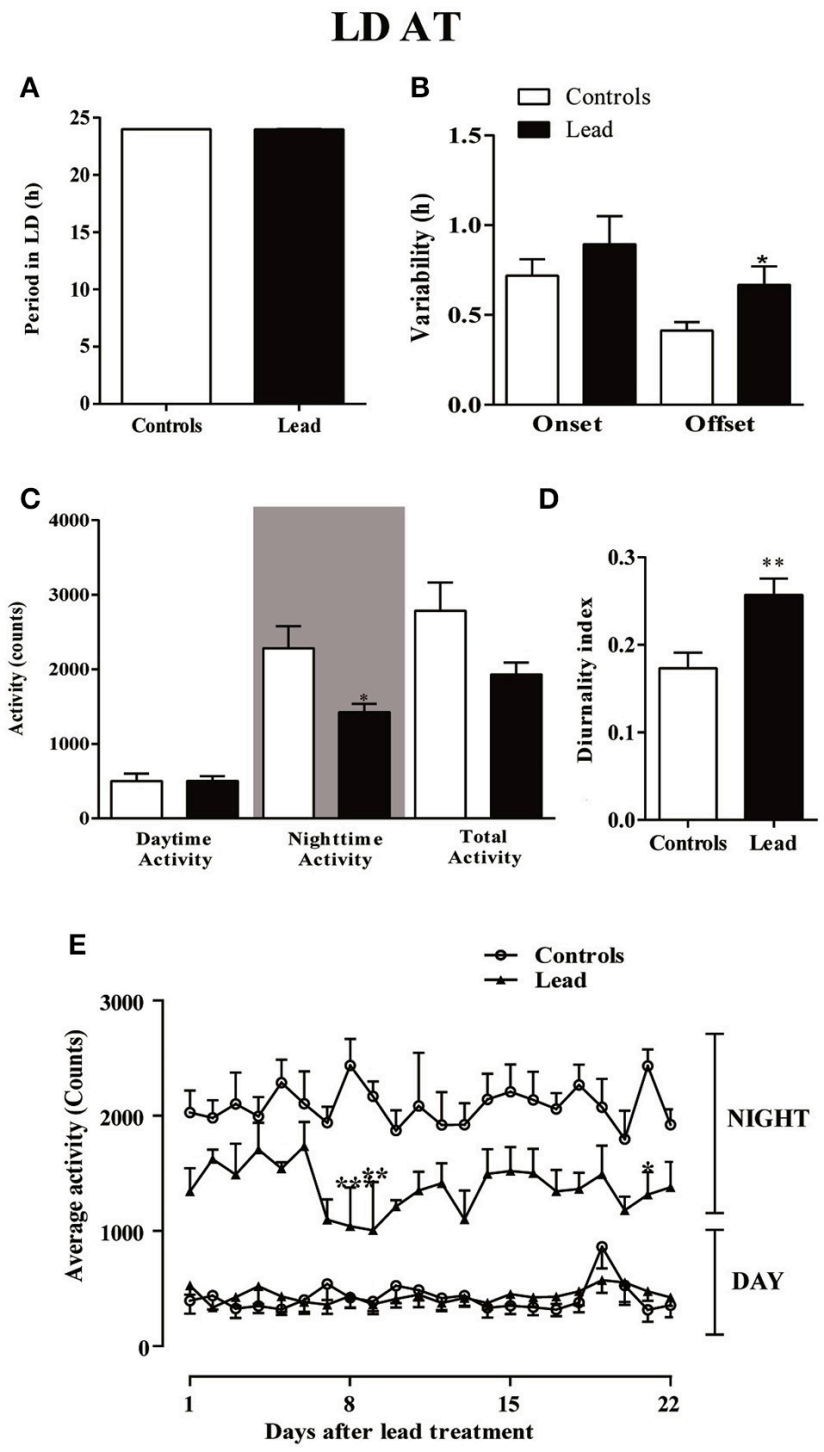
Circadian parameters for controls and lead-intoxicated rats in LD after lead treatment (LDAT). **(A)** Period, **(B)** Onset and offset variability, **(C)** daytime, nighttime and total activity counts, and **(D)** diurnality index. Data from controls (*n* = 7) and lead-intoxicated rats (*n* = 7) were compared using Mann-Whitney test. ^*^P < 0.05, ^**^P < 0.01 in comparison with controls. **(E)** The daily average activity counts from controls (*n* = 7) and lead-intoxicated rats (*n* = 7) were compared using one-way ANOVA followed by Tukey's *post-hoc* test. ^*^P < 0.05, ^**^P < 0.01, ^***^P < 0.001 in comparison with controls.

Three weeks after cessation of lead and sodium acetate injections (LD AT; light on at 00:00), rats showed a strong daily profile of the locomotor activity with a similar period (23.96 ± 0.009 in controls vs. 23.98 ± 0.001 in lead-intoxicated rats; Figure 7A) but a number of parameters were altered. The variability of activity offset was significantly higher in lead-intoxicated rats (0.66 ± 0.103 h vs. 0.41 ± 0.047 h in control rats; *P* < 0.05, Mann Whitney test; Figure 7B), whereas, no difference was observed between the two groups for the variability of activity onset (0.89 ± 0.15 h in lead-intoxicated rats vs. 0.72 ± 0.09 h in control rats; *P* > 0.05, Mann Whitney test, Figure 7B).

A significant decrease in the mean nighttime activity was observed in lead-intoxicated rat (1,425 ± 110.3 vs. 2,282 ± 294.8 in control rats; Figure 7C, *P* < 0.05). No difference was observed in daytime activity (501.1 ± 99.11 in controls vs. 503.1 ± 64.62 in lead-intoxicated rats; Figure 7C). The total activity counts were 2,783 ± 380.7 and 1,928 ± 164.4 in control and lead-intoxicated rats, respectively (Figure 7C) and the diurnality index was significantly increased in lead-intoxicated rats (0.26 ± 0.02) compared with control rats (0.17 ± 0.02) (Figure 7D, Mann Whitney test, *P* < 0.01).

Figure 7E shows the daily change in activity during the light and dark phase under the new LD cycle of rats 3 weeks after lead treatment and the analysis of the daily activity levels (mean activity counts) showed that lead profoundly induced hypoactivity during the dark phase (Two-way repeated measures ANOVA showed a significant effect of treatment [*P* < 0.0001, *F*_(3, 489)_ = 509.6], whereas neither the time nor the interaction (time x treatment) were significant (*P* > 0.05).

**Figure 8:**
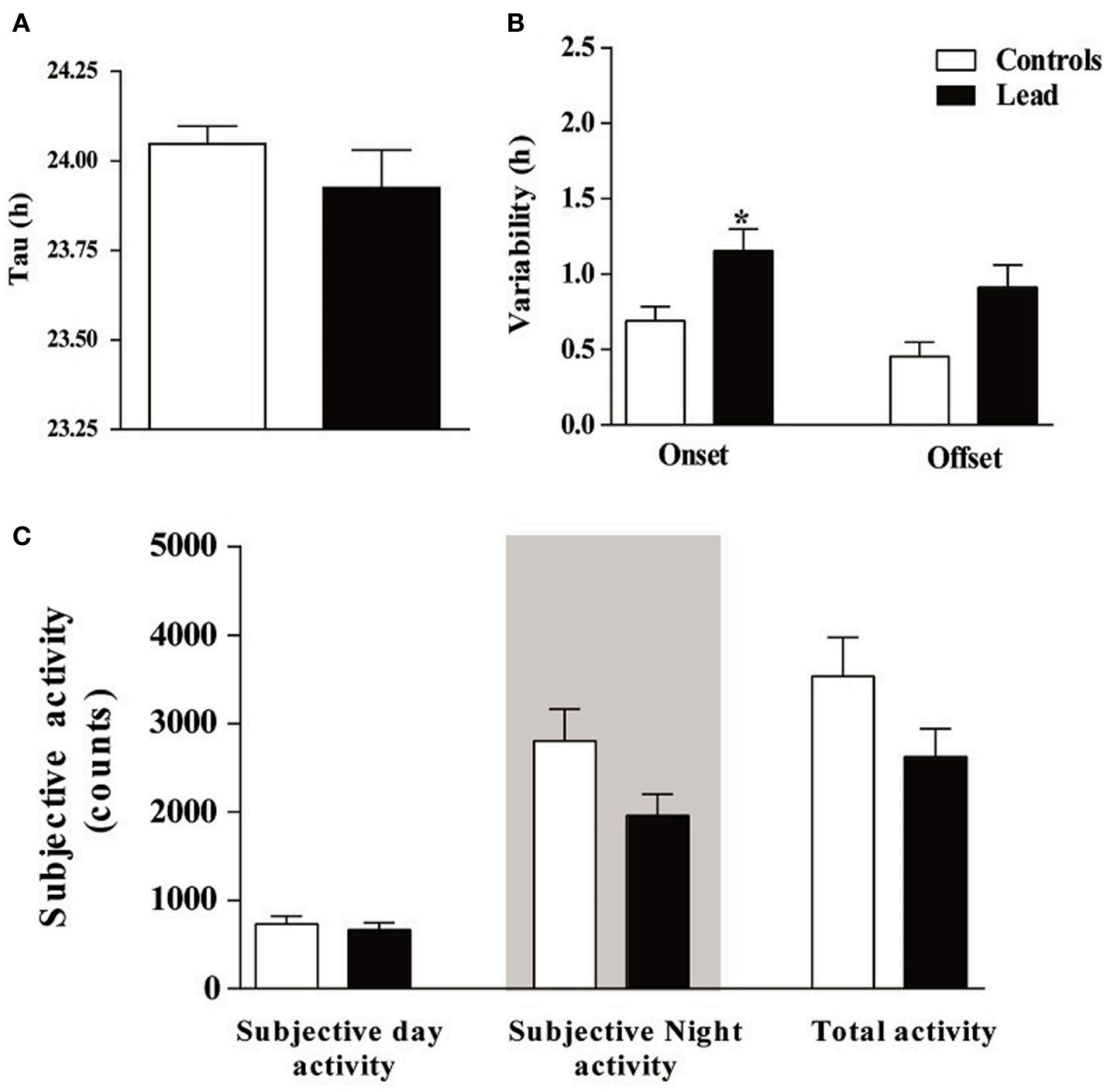
Circadian parameters for controls and lead-intoxicated rats in total darkness (DD) after lead treatment. **(A)** Tau, **(B)** Onset and offset variability and **(C)** subjective activity counts. Data from controls (*n* = 7) and lead-intoxicated rats (*n* = 7) were compared using Mann-Whitney test. ^*^P < 0.05 in comparison with controls.

In order to examine the impact of lead on the circadian clock, we subjected rats to constant conditions of darkness (DD) for 15 days. Under these conditions, lead-intoxicated rats exhibited a locomotor circadian rhythm with a similar free-running period (τ) when compared to controls (23.95 ± 0.038 h in controls vs. 23.93 ± 0.087 h in lead-intoxicated rats; *P* = 0.72, Mann Whitney test; Figure 8A). The activity onsets variability was significantly higher in lead-intoxicated rats (1.15 ± 0.143 h vs. 0.69 ± 0.09 h in controls, Mann Whitney test, *P* < 0.05; Figure 8B) whereas the activity offsets variability was not significantly altered by lead intoxication (0.91 ± 0.148 h vs. 0.45 ± 0.09 h in controls, Mann Whitney test, *P* = 0.084, Figure 8B). Likewise, no differences were found in the activity during the subjective day, in the activity during the subjective night or the total activity (Figure 8C).

### Effect of lead intoxication on the clock proteins immunoreactivity in the SCN

**Figure 9:**
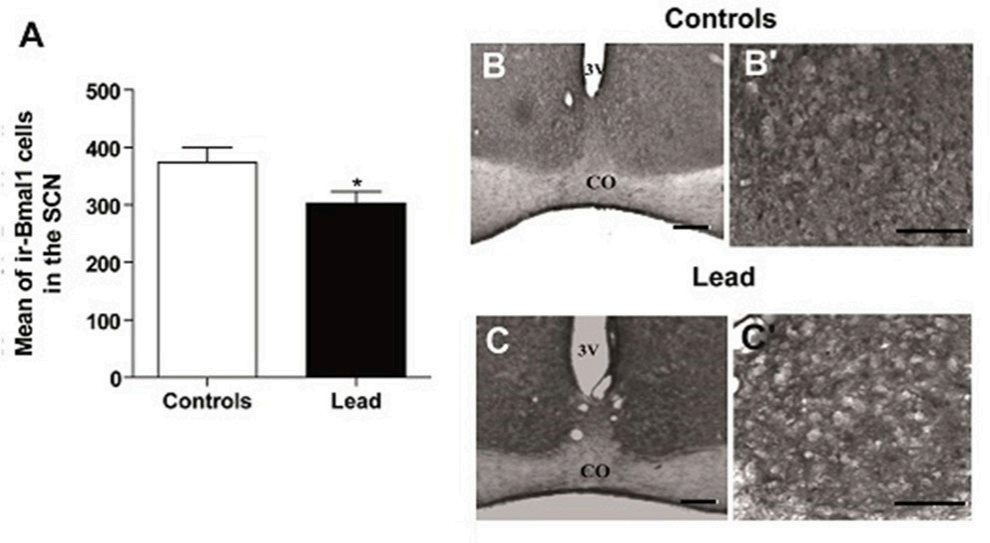
Bmal1-immunoreactive cells in the SCN of controls and lead-intoxicated rats. **(A)** Illustrate histograms of the average number of ir-Bmal1 cells in lead-intoxicated rats compared to controls. **(B,C)** Photomicrographs of Bmal1 immunoreactivity in controls **(B,B**′**)** and lead groups **(C,C**′**)**. Scale bar: **(B,C)** = 200 μm, **(**B′**,C**′**)** = 100 μm. Data from vehicle (*n* = 6) and lead-intoxicated rats (*n* = 6) were compared using the Mann-Whitney test. ^*^P < 0.05 in comparison with controls. OC, Optic chiasm; 3V, Third ventricle.

**Figure 10:**
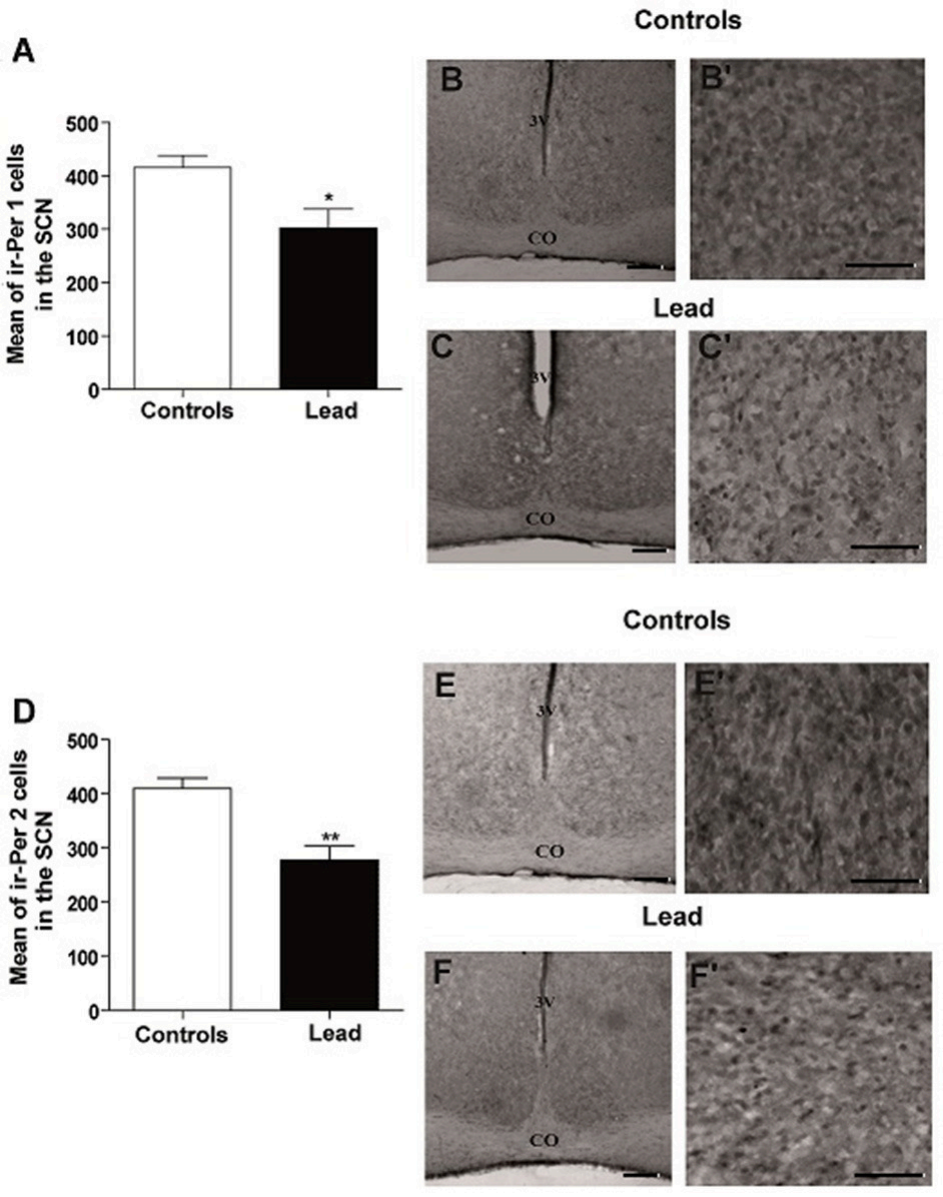
Per1- and Per2- immunoreactive cells in the SCN of controls and lead-intoxicated rats. **(A)** Illustrate histograms of the average number of ir-Per1 cells in lead-intoxicated rats compared to controls. **(B**,**C)** Photomicrographs of Per1 immunoreactivity in controls **(B,B**′**)** and lead groups **(C,C**′**)**. **(D)** Illustrate histograms of the average number of ir-Per2 cells in lead-intoxicated rats compared to controls. **(E,F)** Photomicrographs of Per2 immunoreactivity in controls **(E,E**′**)** and lead groups **(F,F**′**)**. Scale bar: **(B,C,E,F)** = 200 μm, **(B**′**,C**′**,E**′**,F**′**)** = 100 μm. Data from controls (*n* = 6) and lead-intoxicated rats (*n* = 6) were compared using the Mann-Whitney test. ^*^P < 0.05, ^**^P < 0.01 in comparison with controls. OC, Optic chiasm; 3V, Third ventricle.

**Figure 11:**
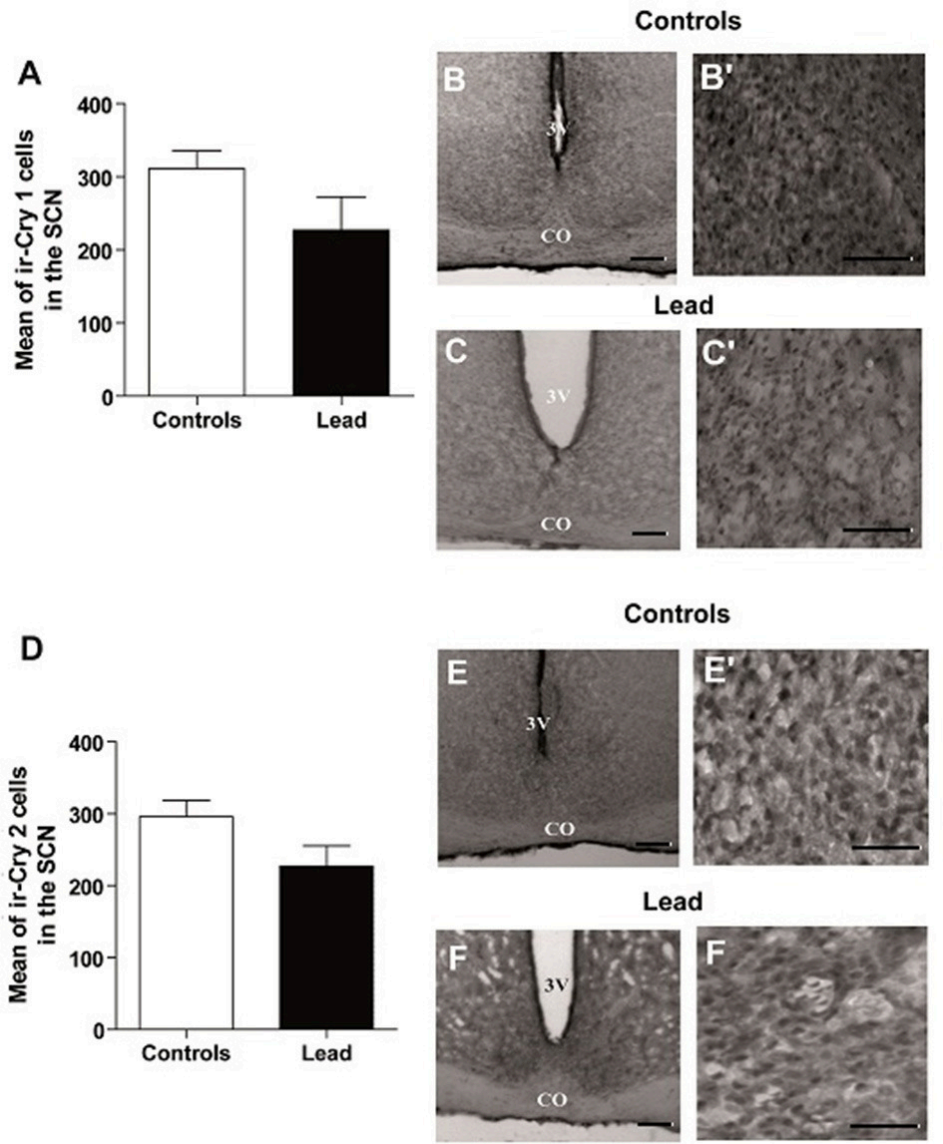
Cry1- and Cry2- immunoreactive cells in the SCN of controls and lead-intoxicated rats. **(A)** Illustrate histograms of the average number of ir-Cry1 cells in lead-intoxicated rats compared to controls. **(B,C)** Photomicrographs of Cry1 immunoreactivity in controls **(B,B**′**)** and lead groups **(C,C**′**)**. **(D)** Illustrate histograms of the average number of ir-Cry2cells in lead-intoxicated rats compared to controls. **(E,F)** Photomicrographs of Cry2 immunoreactivity in controls **(E,E**′**)** and lead groups **(F,F**′**)**. Scale bar: **(B,C,E,F)** = 200 μm, **(B**′**,C**′**,E**′**,F**′**)** = 100 μm. Data from controls (*n* = 6) and lead-intoxicated rats (*n* = 6) were compared using the Mann-Whitney test. OC, Optic chiasm; 3V, Third ventricle.

Figures 9–**11** show the mean number (±SEM) of clock protein immunoreactivity (ir-) in the SCN of lead-intoxicated rats and their respective controls. Lead induced a significant decrease in the mean number of -BMAL1, -PER1, and -PER2 ir-cells in the SCN. In control rats, the average of ir-BMAL1 cells is 374 ± 25.9, whereas, in lead-intoxicated rats, the average of ir-BMAL1 cells is 303.2 ± 19.7 (Mann-Whitney test, *P* < 0.05; Figure 9).

The mean number of ir-PER1 cells is 416 ± 22.72 in controls, whereas, in lead-intoxicated rats, the mean number of ir-PER1 cells is 302.1 ± 35.93 (Mann-Whitney test, *P* < 0.05; Figure 10), and the mean number of ir-PER2 cells is 409.7 ± 19.40 in controls, whereas, in lead-intoxicated rats, the mean number of ir-PER2 cells is 277.7 ± 25.91 (Mann-Whitney test, *P* < 0.05; Figure 10).

In contrast, lead did not affect CRY1 and CRY2 immunoreactivity in the SCN (Figure 11). The number of ir-CRY1 cells is 227 ± 45.3 and 311.1 ± 24.5 in lead-intoxicated and control rats. For CRY2, the number of ir-CRY2 cells is 296 ± 22.7 in controls and 227 ± 28.5 in lead-intoxicated rats.

## Discussion

The main interesting result obtained in this study is the alteration in the locomotor activity rhythm induced by lead toxicity which parallels with a decrease in clock protein expression. Interestingly, these alterations are obtained in all groups of rats that express neurobehavioral dysfunction.

In the current study, we have confirmed in Wistar rat (experiment 1) that lead induces progressive decline in body weight gain, decreased the exploratory activity (number of crossing in open field; Figure 3A) and impaired motor coordination (Figure 3C) as early shown by our previous findings in Sprague dawley rat (Sabbar et al., 2012) and other works (Reiter et al., 1975; Overmann, 1977; Gill et al., 2003; NourEddine et al., 2005; Sansar et al., 2011). Strangely, it seems that in lead-intoxicated rats, the number of rearing in the open filed was not significantly different compared to controls (Figure 3B). However, based on previous data (Shafiq-ur-Rehman et al., 1991), it is more likely that rearing response has decreased the first days following lead intoxication and then increased on day 30. In addition to motor symptoms (Reiter et al., 1975; NourEddine et al., 2005), non-motor symptoms like the rest/activity rhythm is also affected by lead (Collins et al., 1984). In the present study, we have shown for the first time that lead intoxication impaired the locomotor activity rhythm together with a decrease in BMAL1, PER1, and PER2 content in the SCN without inducing any changes in CRY1 and CRY2 content. These results provide strong evidence that lead disturbs circadian function by probably affecting clock protein expression in the SCN.

In the present work, we show that in lead-intoxicated rats, the 24 h rest/activity cycle is fragmented under a 14/10 LD cycle with activity predominantly expressed during the dark phase (Figure 4). Animals showed impaired locomotor activity rhythm consisting of abnormal phasing to the LD cycle, and strong change in the diurnality index (Figure 5E) than those observed in controls. Moreover, lead-intoxicated rats showed also less precision in their daily locomotor activity rhythm as reflected in the increased activity onsets variability (Figure 5F). No difference was however observed in the period of the 24 h locomotor activity rhythm. When the LD cycle was advanced by 6 h, both groups adjusted their daily locomotor activity to the new LD cycle; however, the offset variability was higher in lead-intoxicated rats than controls (Figure 7B). Our results enhance earlier observations reported by Collins et al. (1984) and Shafiq ur Rehman et al. (1986). Collins et al. (1984) showed that in pups chronically exposed to lead for many weeks, the circadian spontaneous locomotor activity was significantly affected, whereas Shafiq ur Rehman et al. (1986) reported that lead intoxication affects circadian rhythm of ambulatory activity. In addition, another study has also demonstrated that lead intoxication affects the circadian patterns of the complex stereotyped behaviors (such as rearing, preening, scratching and biting/licking) (Shafiq ur Rehman, 1999). These alterations disturb the ability of the animal to cope and interact with its environment.

Although no definitive causal links between lead intoxication and PD have been proven, there are a considerable number of epidemiological studies suggested that occupational exposure to specific metals including lead may be a high-risk factor for PD (Gorell et al., 1997; Coon et al., 2006; Weisskopf et al., 2010), we then compared 24 h locomotor activity alterations in lead-intoxicated rats to those observed in PD patients and animal models of PD and reported in the literature. Interestingly, actograms analysis revealed a significant increase in the mean daytime activity during lead intoxication followed by a decrease in the nighttime activity in lead-intoxicated rats, reflecting a poor consolidation of locomotor activity as previously reported (Collins et al., 1984). These findings reproduced those previously reported in PD patients (van Hilten et al., 1994) and in animal models of PD (Ben and Bruguerolle, 2000; Almirall et al., 2001; Boulamery et al., 2010). They strengthen the hypothesis that lead-intoxicated rats lack the ability to maintain robust locomotor activity rhythm under LD cycle, which is similar to the impairment observed in the circadian rhythms of PD patients. In fact, many studies have demonstrated that lower amplitude rest/activity rhythm affected sleep quality (Langmesser et al., 2009; Smith et al., 2009). Indeed, PD patients expressed a number of circadian rhythm alterations such as insomnia and excessive daytime sleepiness (van Hilten et al., 1994; Stevens et al., 2004; Thorpy and Adler, 2005; Ferreira et al., 2006) and nighttime sleep fragmentation (van Hilten et al., 1993; Gunn et al., 2010) strengthening the hypothesis that the alteration in circadian rhythm parameters in lead-intoxicated rats could explain partially sleep/wake cycle disturbances that occur in PD patients (Arnulf et al., 2002). Several parameters in the sleep-wake pattern of animal models of PD have also been described (Fifel et al., 2016). For example, MPTP injection induced a dramatic disruption of sleep/wake architecture associated with reduced (REM) sleep and excessive daytime sleepiness in non-human primate (Barraud et al., 2009). Another study reported a drastic decrease in the latency to the onset of slow wave sleep (SWS) with REM sleep ablation in MPTP- treated rats (Lima et al., 2007). Despite lack of investigations on lead-induced sleep disturbances in human or animals, we found only one report (Kumar and Desiraju, 1992) where the authors showed that lead induced a significant reduction in the delta, theta, alpha and beta band electroencephalogram spectral power in both wakeful and SWS stages. Moreover, our results are in accordance with previous study in PD animal model reporting that ASO (α-synuclein over-expressing) mice did not show neither entrainment deficits to 6 h changes in the LD cycle or, alteration in the locomotor activity rhythm in DD (Kudo et al., 2011). Even that lead-intoxicated rats were able to adjust their locomotor activity rhythm to new LD cycle, the precision of the daily offset of activity was altered, sign of greater fragmentation in activity. All those changes described above and those reported in the studies mentioned previously postulate that lead intoxication may affect the structures and/or functions involved in the circadian timing system. Indeed, anatomical investigations suggested that in addition to other brain region (i.e., hippocampus, cerebellum, retina…), hypothalamus is a target to the neurotoxic action of lead (Wang et al., 2006; Rojas-Castaneda et al., 2011).

In mammals, SCN of the hypothalamus, the main clock pacemaker is involved in the generation and entrainment of circadian rhythms (Meijer and Rietveld, 1989; Lowrey and Takahashi, 2004). The circadian oscillations are generated by molecular mechanism based on feedback loops which is responsible of rhythmic transcription and translation of clock genes (Reppert and Weaver, 2001; Hastings and Herzog, 2004; Lowrey and Takahashi, 2004; Okamura, 2004). *Bmal1* and *Clock* play a key role in feedback loop by acting as a positive regulator; CLOCK-BMAL1 heterodimer is able to induce a rhythmic transcription of other clock genes (Gekakis et al., 1998).

In the present study, we examined the clock protein expression, BMAL1, CRY1, CRY2, PER1, and PER2 in the SCN, and we found that BMAL1, PER1, and PER2 immunoreactivity were significantly declined in lead-intoxicated rats without any changes in CRY1 and CRY2 immunoreactivity in the SCN compared to controls (Figure 11). In this regard, it has been reported that in leukocytes, the expression of BMAL1 was lower in PD patients (Cai et al., 2010). Thus, the decrease in BMAL1 content in the SCN of lead-intoxicated rats could impair the molecular clock by disturbing the transcription factors of other clock genes or clock-controlled output genes. Bunger et al. (2000) showed that under LD, locomotor activity rhythm is impaired and activity levels are reduced in *Bmal1*^−/−^ mice which may explain the alteration of the circadian rhythm of locomotor activity in lead-intoxicated rats. Bunger et al. (2000) demonstrated also that in *Bmal1*^−/−^ mice the expression of *Per1* and *Per2* were very low and not rhythmic. Zheng et al. (1999) provided evidence that *Per1* gene is essential for the functioning of the circadian clock and that *Per2* may regulate *Per1; Per2* mutation leads to a change in the expression of other genes (*Per1*). This mutation displays a shorter circadian period followed by a loss of circadian rhythmicity in constant darkness (Zheng et al., 1999). In DD, lead-intoxicated rats continued to express a rhythmic locomotor activity with a period of approximately 24 h, suggesting that the decrease in PER1 and PER2 contents in the SCN may not be enough to be translated into rhythmicity loss in lead-intoxicated rats. The cryptochrome proteins, CRY1 and CRY2, act as negative regulators in the transcriptional feedback loop (van der Horst et al., 1999; Vitaterna et al., 1999), and inhibit the expression of their own genes and of period genes (Kume et al., 1999). In lead-intoxicated rats, there was no difference in *Cry1* and *Cry2* content in the SCN and this strength the finding that lead-intoxicated rats were rhythmic in total darkness.

In other hand, there is relevant evidence of the implication of several neurotransmitters systems in the sleep/wake regulation/modulation. In parkinsonism, sleep/wake disturbance may result in several neurotransmission failures additionally to DAergic system, i.e., NAergic neurons in the locus coeruleus (Jellinger, 1991; Zarow et al., 2003; Fulceri et al., 2007), and serotonergic neurons in the raphe (Kish, 2003; Kish et al., 2008). Lead is also known to affect neurotransmitter systems, including NA and DA systems (Silbergeld, 1983; Sabbar et al., 2012). We did not measure concentrations of NA or DA following lead intoxication in this study, but we recently find a decrease in striatal DA concentration (unpublished data) and changes in cortical NA concentration in lead-intoxicated rats (same dose and same route of administration; 8). Since NA containing fibers and terminals were demonstrated in the SCN (Cagampang et al., 1994; Jacomy and Bosler, 1995; Vacher et al., 2003), the role of either NA or DA cannot be excluded to explain the circadian rhythm alterations during lead intoxication.

NA might modulate SCN circadian rhythms by regulating the expression of arginine-vasopressin and vasoactive intestinal peptides, two neuropeptides involved in the control of circadian rhythms as previously reported by Vacher et al. (2003). This neurotransmitter might also affect clock genes expression as reported in the astroglial cells of the SCN (Morioka et al., 2010). Furthermore, DA has been shown to modulate the expression of the clock genes (Imbesi et al., 2009) and regulate the BMAL1/CLOCK heterodimer activity (Yujnovsky et al., 2006). A decrease of DA level in the striatum in lead-intoxicated rats (unpublished data) may suggest that this heavy metal may impair the modulatory role that DA exerts and could explain partially the changes in clock protein expression our lead-intoxicated rats. This hypothesis has however to be confirmed by additional experiences.

In conclusion, we have confirmed that lead intoxication induced motor disabilities similar to those reported in animal models of PD. Moreover, we have shown that several 24 h locomotor activity parameters were altered, associated with a decrease in *bmal1, per1*, and *per2* contents in the SCN. Tough, 24 h rest/activity disturbances, have never been extensively explored following lead intoxication, it may be interesting to investigate the mechanism(s) by which lead disrupt circadian rhythmicity, thereby providing evidence that might link lead neurotoxicity to induce Parkinsonism.

## Author contributions

NL designed the experimental protocol. MS collected, analyzed the data, wrote and edited the manuscript. OD assisted with data analysis. NL and AB edited and approved the final draft of the manuscript. All authors read and approved the final manuscript.

### Conflict of interest statement

The authors declare that the research was conducted in the absence of any commercial or financial relationships that could be construed as a potential conflict of interest.
